# Tumors displaying hybrid schwannoma and neurofibroma features in patients with neurofibromatosis type 2 

**DOI:** 10.5414/NP300895

**Published:** 2015-12-28

**Authors:** Blake K. Montgomery, Meghna Alimchandani, Gautam U. Mehta, Ramita Dewan, Cody L. Nesvick, Markku Miettinen, John D. Heiss, Ashok R. Asthagiri, Martha Quezado, Anand V. Germanwala

**Affiliations:** 1 Surgical Neurology Branch, National Institute of Neurological Disorders and Stroke, National Institutes of Health, Bethesda, MD,; 2 Laboratory of Pathology, National Cancer Institute, National Institutes of Health, Bethesda, MD,; 3 Department of Neurological Surgery, University of Virginia School of Medicine, Charlottesville, VA, and; 4 Department of Neurological Surgery, Loyola University School of Medicine, Maywood, IL, USA; *Contributed equally to this work.

**Keywords:** hybrid tumors, peripheral nerve sheath tumor, neurofibromatosis type 2, neurofibroma, schwannoma

## Abstract

Although schwannoma and neurofibroma tumors are generally reported as distinct pathologic diagnoses, sporadic schwannoma/neurofibroma hybrid nerve sheath tumors have been reported in the general population with components of both entities. We report the clinicopathological features of these hybrid nerve sheath tumors in patients with neurofibromatosis type 2 (NF2). A retrospective review of nerve sheath tumor surgical specimens from patients with NF2 enrolled at the National Institutes of Health was performed. Those specimens reported to have schwannoma-like and neurofibroma-like features were selected for further characterization by morphology, immunohistochemical panel (CD34, S100, neurofilament triplet protein (immunostain) (NFTP), epithelial membrane antigen (EMA)), and confirmation as hybrid tumors. Of 43 total NF2 patients undergoing resection of nerve sheath tumors, 11 specimens from 11 (26%) patients were found to be benign nerve sheath tumors exhibiting hybrid features of both neurofibroma and schwannoma. Immunohistochemical studies showed the schwannoma component to be S100+, CD 34- while the neurofibroma component was CD34+, variable S100+. Our experience emphasizes the importance of including this distinct tumor subtype, the schwannoma/neurofibroma hybrid tumor, in the differential diagnosis of nerve sheath tumors in NF2 patients and suggests that the relationship between neurofibroma and schwannoma tumors is closer than previously suspected.

## Introduction 

Neurofibromatosis type 2 (NF2) is an autosomal dominant disease that is caused by loss-of-function mutations in the *NF2* gene located on chromosome 22q12.2. NF2 occurs in ~ 1 in 40,000 people [1]. The *NF2* mutation limits the function of the tumor-suppressor protein, merlin (schwannomin), which regulates proliferation of many cell types of neural crest lineage. 

Patients with NF2 are at increased risk of developing schwannomas, meningiomas, and gliomas, such as astrocytomas [2]. Schwannomas are the most common type of tumor seen in NF2. Neurofibromas have rarely been described in the literature in association with NF2 and are more commonly seen in neurofibromatosis type 1 (NF1) [3]. Unlike schwannomas, these tumors arise within the neural fascicles. 

Although sporadic hybrid schwannoma/neurofibroma tumors have been described, schwannoma/neurofibroma hybrid tumors in the setting of NF2 have rarely been reported in the literature, and their pathology has not been fully described [4, 5, 6, 7, 8, 9]. We report a series of patients with NF2 harboring schwannoma-neurofibroma hybrid nerve sheath tumors. 

## Materials and methods 

A retrospective review of all resected nerve sheath tumor specimens from patients with NF2 enrolled in the Natural History Protocol #08-N-0044 at the Clinical Center, National Institutes of Health between 2007 and 2013 was performed. Pathologists (MQ, MM, MA) selected formalin fixed paraffin-embedded (FFPE) tissue of surgical specimens that, on original histological review, had features of both schwannoma and neurofibroma. The FFPE tissue was sectioned and immunohistochemical staining was performed using a panel of antibodies consisting of CD34, S100, neurofilament triplet protein (immunostain) (NFTP), and epithelial membrane antigen (EMA). Immunohistochemical procedures were performed using an automated immunostainer following the manufacturer’s specifications. Specimens displaying immunohistochemical features of both schwannoma and neurofibroma were then confirmed as schwannoma/neurofibroma hybrid tumors. Further analyses of patients with hybrid tumors were then performed, including recording patient demographics, tumor location, clinical and radiographic features, and long-term follow-up. Clinical notes, intraoperative reports, and imaging of the selected patients were analyzed. 

## Results 

43 patients with NF2 underwent surgery for removal of one or more nerve sheath tumors. 28 patients had surgery for non-vestibular intracranial schwannomas or peripheral nerve sheath tumors, 10 patients had surgery for vestibular schwannomas, and 5 patients had surgery for both vestibular and non-vestibular schwannomas. On microscopic evaluation, 11 specimens from 11 (26%) patients had benign nerve sheath tumors exhibiting hybrid features of both neurofibroma and schwannoma. The 11 patients had a median age of 35 years (range 12 – 52 years) and included 7 females and 4 males. The tumors were most commonly located in the extremities, with some involving the head, neck, and trunk, with size varying from 0.3 cm to 8.4 cm in diameter. These clinicopathological features have been summarized in Table 1. 

Eight of these patients presented with pain while 3 developed neurologic symptoms related to tumor growth. Eight patients had multiple contiguous tumor nodules noted on imaging. Gross total resection was achieved in 10 cases and no intraoperative complications occurred. Patients received post-operative clinical and radiographic follow-up for an average of 36 months (6 – 118 months). No gross signs of recurrence or delayed complications occurred during follow-up. Symptomatic relief was noted in all cases. 

### Imaging findings

**Figure 1.**
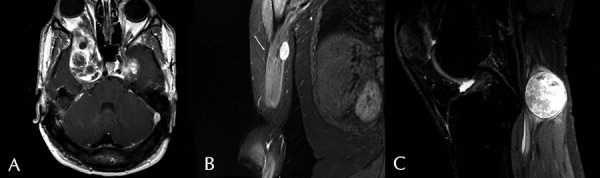
Post-contrast T1 weighted MR image. A: Axial image displaying large enhancing tumors of the cavernous sinus and right orbit (Patient 11). B: Coronal image of right arm showing enhancing tumor (Patient 4). C: Sagittal image of right knee depicting enhancing tumor on right tibial nerve (Patient 3).

T1-weighted magnetic resonance images with contrast were reviewed for all patients. All of the tumors were enhancing (Figure 1). Measurements are listed in Table 1. 

### Microscopic findings

**Figure 2.**
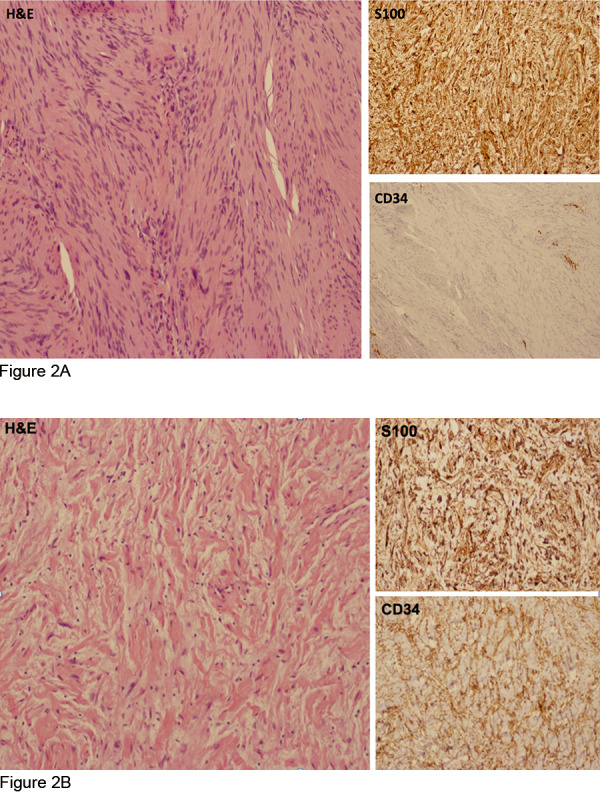
A hybrid nerve sheath tumor exhibits dual features of cellular schwannoma (A) with spindle cells S100+, CD34-, adjacent to areas of neurofibroma (B) showing lower cellularity with wavy nuclei in a collagenous stroma and cells S100+, CD34+.

Pathology review of these 11 specimens showed dual histologic components of both schwannoma and neurofibroma (Table 2). Schwannoma histology, incorporating highly ordered cellular components (Antoni A) with loose, myxoid components (Antoni B) were the distinctive features with a minor component demonstrating neurofibroma-like features [10]. Areas of neurofibroma had wavy nuclei interspersed with collagen fibers. Degenerative changes including focal cellular atypia, hyalinization of blood vessels and aggregation of histiocytes were variably present. Tumors were further characterized by immunohistochemical studies with S100, CD34, NFTP, and EMA. Scattered cellular atypia was present in a few sections but mitotic activity was very low and necrosis absent. The observed dual histology of these hybrid tumors was further supported by a corresponding dual immunophenotype: the schwannoma component was S100 positive and CD34 negative while the neurofibroma component contained CD34 positive fibroblasts and S100 protein-positive Schwann cells (Figure 2A, B). Immunostain for NFTP highlighted residual axonal processes in the neurofibroma component. EMA was negative in both components, showing no evidence for a perineurioma in these hybrid tumors. However, an EMA-positive perineurioma was removed from the finger of 1 patient who also had a separate hybrid schwannoma/neurofibroma. 

## Discussion 

Schwannomas were first described in the early 1900s by Verocay and Antoni [11, 12]. NF2 schwannomas are most commonly located on the vestibular nerve (bilateral vestibular schwannomas), but can also manifest on other cranial nerves, spinal nerves, and peripheral nerves [13, 14, 15, 16]. On gross examination these tumors are encapsulated by epineurium and on cut section can vary in color from pink to yellow white [10]. Histologically, schwannomas contain two distinct components. Antoni A areas consist of compact cellular regions consisting of nuclear palisading, cell whorling, and Verocay bodies (areas with rows of nuclei separated by fibrillary cell processes) while Antoni B areas represent hypocellular areas with less order and a more loosely structured matrix [10, 17, 18]. Degenerative changes may be seen in Antoni B areas with hemosiderin-laden histiocytes and collagenous fibrosis [17]. Immunohistochemical studies show that schwannomas stain positive for S100 in both the nucleus and the cytoplasm [17, 19]. S100 stains both the Antoni A and B portions of the tumor, with more intense staining of the Antoni A regions [19]. The tumor itself is typically negative for EMA, but EMA positive perineurial cells are common within the capsule [17]. CD34 positivity may be seen in the periphery of the tumor. 

Neurofibromas arise from cutaneous and deep nerves, autonomic nerves, and spinal nerve roots and typically exhibit one of three growth patterns: localized, diffuse, or plexiform [10]. Neurofibromas consist of Schwann cells, fibroblasts, histiocytes, and mast cells and may also contain perineurial cells [20]. The tumors commonly incorporate axons throughout a nerve, making complete surgical tumor resection without nerve sacrifice impossible [21, 22]. Histologically neurofibromas have smaller wavy nuclei, ~ 1/3 – 1/2 size of the nuclei seen in schwannomas. Immunohistochemical studies show neurofibromas to have CD34 positivity, variable S100 positivity, and rare EMA positivity if perineurial cells are present [23]. 

In a smaller study, hybrid schwannoma/neurofibroma peripheral nerve sheath tumor was found in 10 of 14 (71%) patients with schwannomatosis [7]. Excluding patients with this diagnosis revealed that 26% of the remaining hybrid tumors occurred in patients with NF2. In our study, there was a 26% incidence of schwannoma/neurofibroma hybrid tumors in patients diagnosed with NF2 that underwent surgical resection. These so-called hybrid tumors represent a rare, but distinct entity affecting the NF2 patient population. Hybrid tumors were located in the peripheral nervous system in all 11 patients. The majority of patients had multiple contiguous tumor nodules on imaging. Tumor size varied greatly among patients. Schwannoma histology predominated, with neurofibroma histology comprising a minor component of the hybrid tumors. The observed dual histology of these hybrid tumors was further supported by a corresponding dual immunophenotype: schwannoma component stained S100 positive and CD 34 negative while neurofibroma component stained CD34 positive with variable S100 expression. 

Our findings indicate that hybrid tumors having dual morphologic and immunophenotypic features of schwannoma/neurofibroma may be more prevalent in the NF2 population than previously realized. Increased awareness of this entity will lead to better estimates of its true prevalence. A needle biopsy sample containing histologic features of schwannoma or neurofibroma alone does not exclude a diagnosis of hybrid tumor, because such a small biopsy could sample a single tumor type and miss the other component of a hybrid tumor. 

## Conclusions 

Our experience suggests that the derivation of schwannomas and neurofibromas may be less distinct than classically appreciated. Although Schwann cells are the suspected tumor cell of origin in both cases, the finding of a hybrid pathology supports the notion that these entities may indeed rather represent a spectrum of Schwann cell pathology. The schwannoma/neurofibroma hybrid tumor should be included in the differential diagnosis of nerve sheath tumors in patients with NF2. Surgical resection of these tumors can be performed safely and effectively with low rates of recurrence. Future study of the genetics of this tumor subtype is warranted. 

## Acknowledgment 

This research was supported by the Intramural Research Program of the National Institute of Neurological Disorders and Stroke at the National Institutes of Health (NIH) and through the NIH Medical Research Scholars Program, a public-private partnership supported jointly by the NIH and generous contributions to the Foundation for the NIH from Pfizer Inc., The Doris Duke Charitable Foundation, The Alexandria Real Estate Equities, Inc. and Mr. and Mrs. Joel S. Marcus, and the Howard Hughes Medical Institute, as well as other private donors. 

## Conflict of interest 

The authors report no conflict of interest concerning the materials or methods used in this study or the findings specified in this paper. 

**Table 1.** Patient demographics and pathologic characteristics of eleven schwannoma-neurofibroma hybrid tumors included in the study.

Case #	Age (y)	Gender	Tumor location	Single or multiple tumor nodules	Tumor size (cm)	Additional features
1	52	F	Ulnar nerve	Single	2	Focal atypia
2	17	M	Scalp, finger, posterior neck	Multiple	1 – 3	Plexiform growth pattern, Verocay bodies
3	49	F	Tibial nerve	Single	4.2	Verocay bodies, hyalinized blood vessels, focal hemosiderin
4	26	F	Right arm	Multiple (at least 10)	0.4 – 8.4	Verocay bodies
5	12	F	Hard palate, finger	Multiple	1 – 2	Verocay bodies
6	45	M	Paravertebral	Multiple	1 – 2.7	Granulomatous inflammation
7	24	F	Cranial nerve XI	Multiple	0.3 – 0.5	
8	44	F	Leg, finger	Multiple	0.3 – 4.3	Separate perineurioma was also found
9	29	M	Forearm	Single	1.5	
10	47	F	Peri-scapular	Multiple	4 – 6	
11	35	M	Orbit, nasal turbinates	Multiple	1- 2.3	Focal myxoid stroma

**Table 2.** Typical histological features of benign nerve sheath tumors: schwannoma and neurofibroma.

	Schwannoma	Neurofibroma
Cellularity	Moderate to high	Low to moderate
Cytology	Spindle cells with extensive nuclear palisading and Verocay bodies (Antoni A areas) alternating with hypocellular, loosely myxoid regions (Antoni B areas)	Wavy nuclei, focal nuclear atypia
Immunophenotype	S100+; CD34-	CD34+; S100 +/– (variable); NFTP stains residual axonal processes
Additional features	Hyalinized vessels with occasional thrombosis; degenerative areas may be histiocyte-rich with hemosiderin representing ischemic infarction	Collagenous background stroma may be myxoid

## References

[1] Evans DG Moran A King A Saeed S Gurusinghe N Ramsden R Incidence of vestibular schwannoma and neurofibromatosis 2 in the North West of England over a 10-year period: higher incidence than previously thought. Otol Neurotol. 2005; 26: 93–97. 15699726 10.1097/00129492-200501000-00016

[2] Asthagiri AR Parry DM Butman JA Kim HJ Tsilou ET Zhuang Z Lonser RR Neurofibromatosis type 2. Lancet. 2009; 373: 1974–1986. 19476995 10.1016/S0140-6736(09)60259-2 PMC4748851

[3] Evans DG Huson SM Donnai D Neary W Blair V Newton V Strachan T Harris R A genetic study of type 2 neurofibromatosis in the United Kingdom. II. Guidelines for genetic counselling. J Med Genet. 1992; 29: 847–852. 1479599 10.1136/jmg.29.12.847 PMC1016199

[4] Dubuisson A Fissette J Vivario M Reznik M Stevenaert A A benign tumor of the sciatic nerve: case report and review of the literature. Acta Neurol Belg. 1991; 91: 5–11. 2028739

[5] Feany MB Anthony DC Fletcher CD Nerve sheath tumours with hybrid features of neurofibroma and schwannoma: a conceptual challenge. Histopathology. 1998; 32: 405–410. 9639114 10.1046/j.1365-2559.1998.00419.x

[6] Halliday AL Sobel RA Martuza RL Benign spinal nerve sheath tumors: their occurrence sporadically and in neurofibromatosis types 1 and 2. J Neurosurg. 1991; 74: 248–253. 1846409 10.3171/jns.1991.74.2.0248

[7] Harder A Wesemann M Hagel C Schittenhelm J Fischer S Tatagiba M Nagel C Jeibmann A Bohring A Mautner VF Paulus W Hybrid neurofibroma/schwannoma is overrepresented among schwannomatosis and neurofibromatosis patients. Am J Surg Pathol. 2012; 36: 702–709. 22446939 10.1097/PAS.0b013e31824d3155

[8] Murărescu ED Ivan L Mihailovici MS Neurofibroma, schwannoma or a hybrid tumor of the peripheral nerve sheath? Rom J Morphol Embryol. 2005; 46: 113–116. 16286996

[9] Youens KE Woodward J Wallace D Cummings TJ Hybrid neurofibroma-schwannoma of the orbit. Orbit. 2008; 27: 223–225. 18569835 10.1080/01676830802009804

[10] Goldblum JR Weiss SW Folpe AL Enzinger & Weiss‘s Soft Tissue Tumors. 6th ed. Philadelphia: Elsevier; 2013.

[11] Antoni NR Über Rückenmarkstumoren und Neurofibrome. München & Wiesbaden: Bergmann; 1920.

[12] Verocay J Zur Kenntnis der „Neurofibrome“. Beitr Pathol Anat Allg Pathol.. 1910; 48: 1–69.

[13] Fisher LM Doherty JK Lev MH Slattery WH Distribution of nonvestibular cranial nerve schwannomas in neurofibromatosis 2. Otol Neurotol. 2007; 28: 1083–1090. 18043434 10.1097/MAO.0b013e31815a8411

[14] Hoa M Slattery WH Neurofibromatosis 2. Otolaryngol Clin North Am. 2012; 45: 315–332. 22483819 10.1016/j.otc.2011.12.005

[15] Mautner VF Lindenau M Baser ME Hazim W Tatagiba M Haase W Samii M Wais R Pulst SM The neuroimaging and clinical spectrum of neurofibromatosis 2. Neurosurgery. 1996; 38: 880 885–885 886. 8727812 10.1097/00006123-199605000-00004

[16] Parry DM Eldridge R Kaiser-Kupfer MI Bouzas EA Pikus A Patronas N Neurofibromatosis 2 (NF2): clinical characteristics of 63 affected individuals and clinical evidence for heterogeneity. Am J Med Genet. 1994; 52: 450–461. 7747758 10.1002/ajmg.1320520411

[17] Miettinen M Modern Soft Tissue Pathology: Tumors and Non-Neoplastic Conditions (Cambridge Medicine) 1st ed. Cambridge: Cambridge University Press; 2010.

[18] Wippold FJ Lubner M Perrin RJ Lämmle M Perry A Neuropathology for the neuroradiologist: Antoni A and Antoni B tissue patterns. AJNR Am J Neuroradiol. 2007; 28: 1633–1638. 17893219 10.3174/ajnr.A0682 PMC8134199

[19] Sharma S Sarkar C Mathur M Dinda AK Roy S Benign nerve sheath tumors: a light microscopic, electron microscopic and immunohistochemical study of 102 cases. Pathology. 1990; 22: 191–195. 1708860 10.3109/00313029009086659

[20] Scheithauer BW Woodruff JM Erlandson R Tumors of the Peripheral Nervous System. Washington, DC: Armed Forces Institute of Pathology; 1997.

[21] Smith TW Bhawan J Tactile-like structures in neurofibromas. An ultrastructural study. Acta Neuropathol. 1980; 50: 233–236. 6774592 10.1007/BF00688760

[22] Waggener JD Ultrastructure of benign peripheral nerve sheath tumors. Cancer. 1966; 19: 699–709. 4160262 10.1002/1097-0142(196605)19:5<699::aid-cncr2820190516>3.0.co;2-h

[23] Zamecnik M Michal M Perineurial cell differentiation in neurofibromas. Report of eight cases including a case with composite perineurioma-neurofibroma features. Pathol Res Pract. 2001; 197: 537–544. 11518046 10.1078/0344-0338-00124

